# Diffusion Model for DAS-VSP Data Denoising

**DOI:** 10.3390/s23208619

**Published:** 2023-10-21

**Authors:** Donglin Zhu, Lei Fu, Vladimir Kazei, Weichang Li

**Affiliations:** Aramco Americas—Houston Research Center, Houston, TX 77084, USA; dzhu@mines.edu (D.Z.); vladimir.kazei@aramcoamericas.com (V.K.); weichang.li@aramcoamericas.com (W.L.)

**Keywords:** distributed acoustic sensing (DAS), vertical seismic profiling (VSP), denoising, diffusion model

## Abstract

Distributed acoustic sensing (DAS) has emerged as a transformational technology for seismic data acquisition. However, noise remains a major impediment, necessitating advanced denoising techniques. This study pioneers the application of diffusion models, a type of generative model, for DAS vertical seismic profile (VSP) data denoising. The diffusion network is trained on a new generated synthetic dataset that accommodates variations in the acquisition parameters. The trained model is applied to suppress noise in synthetic and field DAS-VSP data. The results demonstrate the model’s effectiveness in removing various noise types with minimal signal leakage, outperforming conventional methods. This research signifies diffusion models’ potential for DAS processing.

## 1. Introduction

Distributed acoustic sensing (DAS) has seen widespread adoption as an emerging technology for seismic signal recording, presenting notable advantages over conventional geophones [1]. Factors such as reduced costs, a wider detection range, and enhanced spatial resolution render DAS an attractive choice in modern seismic studies [2,3]. Despite these benefits, the implementation of DAS, and in particular, vertical seismic profile data (VSP), introduces its own set of unique challenges that require careful navigation.

**Figure 1:**
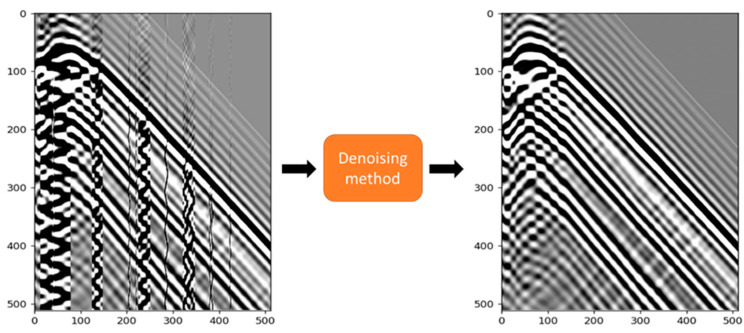
Denoising seismic data task. Noisy seismic (input) is mapped to clean seismic shot gather (label).

One of the predominant challenges encountered in DAS-VSP data processing is the presence of noise from a diverse range of sources. Environmental noise, horizontal and fading noise, poor coupling, and flow interference constitute significant impediments that can severely impact the accuracy and efficacy of subsequent imaging, interpretation, and analysis of seismic data [1,4]. The mitigation of these noise types and the improvement of the signal-to-noise ratio (SNR) have thus become critical aspects of DAS-VSP data processing. Here, we focus on the elimination of “zigzag” noise [5] typical for DAS data and mainly attributed to coupling (Figure 1). Consequently, the development and application of efficient denoising methods represents a crucial research area in contemporary seismic studies.

Various denoising methods have been deployed for traditional seismic data processing. Techniques such as wavelet transform [6], band-pass filtering [7], and f-x deconvolution [8] have demonstrated considerable potential in enhancing the SNR in the presence of random noise. For high-amplitude erratic noises such as swell noises, robust singular spectrum analysis [9] and its adaptive version [10] recover signals from the low-rank components of the transformed data while removing erratic noise components via soft-thresholding. Horizontal noises in DAS data are typically addressed by stacking all or a significant portion of the DAS record and subtracting this estimate from each trace in the DAS record [5,11,12]. However, the efficacy of these conventional methods varies considerably with different types of noise. In particular, they have shown limited success in dealing with noise types such as fading noise and poor coupling [4,5,13]. Poor coupling in DAS data is known to lead to zigzag noise in correlated data [5]; we focus our attention on eliminating this type of noise (Figure 1). 

The limitations of these conventional methods necessitate the exploration of alternative, more advanced strategies. The rise of deep learning has presented new opportunities in this context, with convolutional neural networks (CNNs) showing remarkable potential in seismic denoising applications [14,15]. CNN-based denoising methods leverage the power of machine learning, utilizing complex architectures and training algorithms to tackle the intricacies of seismic noise. The convolutional neural network (CNN) has been explored for seismic data denoising [14,16]. More recent work [17] in combining physics surrogate constraint with a CNN type of architect has demonstrated its effectiveness in suppression of severe coherent noises such as ground roll. The CNN-based denoising methods have shown the power to deal with complex noise in DAS data [18,19].

However, CNN-based methods are not without their limitations. Most ML-based methods rely on supervised learning algorithms which means ground truth is needed for training (Figure 1). It is hard to simulate the specific noise types in DAS-VSP data. One way to obtain noise data is extracting it from field data prior to first signal arrivals. But it is inevitable to introduce seismic signals with noise extraction if the noise is not easily separated from the signal in space and time. Moreover, when denoised field data derived from conventional methods serve as training datasets, the CNN models may inherit the limitations of these methods, making it difficult for the model to exceed the performance of the techniques used to create the training data. 

Given these constraints, researchers have begun exploring alternative deep learning strategies, particularly generative models such as diffusion models [20]. Diffusion models present several advantages over other generative models, like generative adversarial networks (GANs). They are simpler to train and do not suffer from issues like mode collapse or the generation of low-quality outputs, which are common in GANs [21,22]. Durall et al. [23] demonstrated the capability of diffusion models for seismic processing from demultiple to interpolation. However, despite their potential, diffusion models remain underexplored in the context of DAS-VSP processing.

This study introduces the use of diffusion models for DAS-VSP noise suppression, presenting a pioneering approach in this domain. First, we generate a volume of synthetic DAS-VSP training data using forward modeling. We manipulate various parameters in this process, such as source location and the main frequency of the wavelet, creating a rich, diverse dataset for training the diffusion model.

Subsequently, we apply the trained diffusion model to denoise specific types of noise present in DAS-VSP data. The experimental results from both the synthetic and field experiments show that our proposed workflow effectively suppresses various types of noise with minimal impact on the effective DAS signals. Furthermore, the diffusion model exhibits a greater tolerance to the variety in noise types, demonstrating its robustness and versatility.

This study positions diffusion models as a promising architecture for future research in DAS-VSP noise suppression. By overcoming the limitations of conventional methods and the restrictions of existing CNN-based techniques, the use of diffusion models represents a significant step forward in seismic data processing. As we continue to refine this method and expand its applications, we expect to see a substantial improvement in the quality of DAS-VSP data, contributing significantly to the broader field of seismic studies.

## 2. Methods

This study proposes a denoising workflow for DAS-VSP data leveraging the power of diffusion models [20], more specifically, the denoising diffusion probabilistic models (DDPMs) [24]. Diffusion models use a Markov chain, which progressively transmutes one distribution into another, an idea employed in non-equilibrium statistical physics [25] and sequential Monte Carlo [26].

In this context, a DDPM is a parameterized Markov chain, trained through variational inference, to generate samples which, after a finite time, match the data. The transitions of this chain are designed to reverse a diffusion process—a Markov chain adding noise to the data in small increments until the original signal is completely masked. The process of transitioning to conditional Gaussians when the diffusion consists of small amounts of Gaussian noise allows for a notably straightforward neural network parameterization.

**Figure 2:**
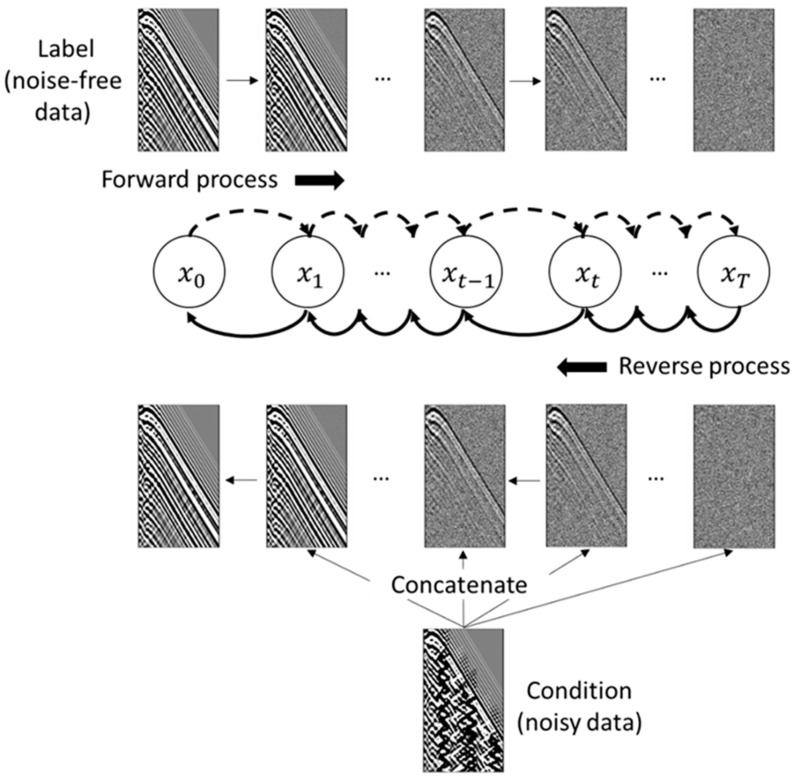
Workflow of proposed diffusion model for DAS-VSP data denoising. The forward process is untrainable (adding noise only), and the reverse process contains learnable parameters.

The process is illustrated in Figure 2 and can be divided into two parts: the forward process and the reverse process. We further extend this by including a section on the conditional diffusion models.

### 2.1. Forward Process

In the forward process, devoid of learnable parameters, the concluding distribution morphs into an isotropic-independent Gaussian distribution as time increases. It stipulates the real data distribution, $x_{0}\sim q(x_{0})$, and introduces minor Gaussian noise cumulatively over T steps. This process results in a sequence of samples $x_{1},x_{2},\ldots ,x_{T}$, each perturbed with additional noise. The noise’s mean and variance are governed by a factor, β, within [0, 1], and follows an ascending sequence, $\beta_{1}<\beta_{2}<\ldots <\beta_{T}$, indicating an increment in the noise added over time. The forward process $q(x_{t}|x_{t-1})$ is defined by:

(1) $$q\left(x_{t}|x_{t-1}\right)=N(x_{t};\sqrt{1-\beta_{t}}x_{t-1},\beta_{t}I),$$

where N denotes normal distribution, I represents the unit matrix. Following the Markov chain principle, the joint probability distribution of $x_{1:T}$ given $x_{0}$ is:

(2) $$q\left(x_{1:T}|x_{0}\right)=\prod_{t=1}^{T}q\left(x_{t}|x_{t-1}\right).$$

### 2.2. Reverse Process

The reverse process of the diffusion model is essentially the denoising process. In this process, we aim to recover the original distribution of $x_{0}$ from the standard Gaussian distribution $x_{T}\sim N(0,I)$. If $q(x_{t-1}|x_{t})$ can be gradually obtained, it would still be a Gaussian distribution, assuming $q\left(x_{t}|x_{t-1}\right)$ follows a Gaussian distribution and $\beta_{t}$ is small enough [27]. Every step of the reverse process $p_{\theta }(x_{t-1}|x_{t})$ can be defined by:

(3) $$p_{\theta }\left(x_{t-1}|x_{t}\right)=N(x_{t-1};\mu_{\theta }\left(x_{t},t\right),\Sigma_{\theta }\left(x_{t},t\right)),$$

where θ represents the parameters learned by the neural network. The reverse process is defined by:

(4) $$p_{\theta }\left(x_{0:T}\right)=p(x_{T})\prod_{t=1}^{T}p_{\theta }\left(x_{t-1}|x_{t}\right).$$

The neural network used in the reverse process to simulate the distribution $p_{\theta }\left(x_{t-1}|x_{t}\right)$ is the U-Net [28]—a classic choice for image processing without changing the image shape that combines convolutional layers of a standard autoencoder architecture with skip connections to regularize the outputs. The U-Net is enhanced by adding Resblocks [29] introducing the differentiation capability directly into the network—thus allowing the focus on noise and attention layers [30] spreading the receptive field of the network. Adding Resblocks and attention mechanisms to the U-Net leads to improved performance by allowing the training of deeper models, improving feature learning, and enhancing focus on the most relevant parts of the input [23,24,28].

### 2.3. Conditional Diffusion Models

As a significant extension of the DDPM, conditional diffusion models [31,32] have been introduced to guide the diffusion process in a specific way, informed by the certain conditioning variable, y. The forward process now depends on these variables and is given by:

(5) $$q\left(x_{t}|x_{t-1},y\right)=N(x_{t};\sqrt{1-\beta_{t}}x_{t-1},\beta_{t}I).$$

Similarly, the reverse process now also takes these conditioning variables into account:

(6) $$p_{\theta }\left(x_{t-1}|x_{t},y\right)=N(x_{t-1};\mu_{\theta }\left(x_{t},y,t\right),\Sigma_{\theta }\left(x_{t},y,t\right)).$$

### 2.4. Training and Loss Function

Training the diffusion models and obtaining the corresponding parameter θ is performed by optimizing the negative log-likelihood:

(7) $$L={Ε}_{q(x_{0})}\left[-logp_{\theta }(x_{0})\right].$$

After applying the variational lower bound (VLB) [33,34] and the reparameterization trick [34], the loss function simplifies to:

(8) $$L_{t}={Ε}_{{t,x}_{0},\;\theta }\left[{\left\|\epsilon -\epsilon_{\theta }\left(\sqrt{{\bar{\alpha }}_{t}}x_{0}+\sqrt{1-{\bar{\alpha }}_{t}}\epsilon ,t\right)\right\|}^{2}\right],$$

where $\epsilon_{\theta }$ derives from the network, ϵ represents the Gaussian noise, $\alpha_{t}=1-\beta_{t}$, and ${\bar{\alpha }}_{t}=\prod_{i=1}^{t}\alpha_{i}$.

Training the conditional diffusion model focuses on the condition variables y, with the loss function now being:

(9) $$L_{t}={Ε}_{{t,x}_{0},y,\;\theta }\left[{\left\|\epsilon -\epsilon_{\theta }\left(\sqrt{{\bar{\alpha }}_{t}}x_{0}+\sqrt{1-{\bar{\alpha }}_{t}}\epsilon ,t,y\right)\right\|}^{2}\right]$$

This approach enables the model to generate samples that closely match a specific data distribution dictated by the conditioning variables. The complexity added by the conditioning variables necessitates more nuanced training, making the process challenging yet yielding more precise results. The hyperparameters of the model were largely adopted from [23,24] and empirically fine-tuned for best performance. We observed that larger patch sizes substantially improved performance, yet to balance the GPU loads and dataset size we stopped at 512 × 512 patches. The final hyperparameters are listed in Table 1. The model was trained using 3 Nvidia A100 GPUs.

### 2.5. Synthetic Training Data Generation

Based on the analysis of the signal dominant frequent, velocity, wavelet, and noise type of the DAS-VSP data, we constructed synthetic data for training and testing the proposed conditional diffusion model. The synthetic dataset consisted of two parts, the clean set and the noisy set. For simplicity of the synthetic forward modeling, we considered the modeling with acoustic wave propagation. The acoustic solver simulated the pressure wavefield which for our manuscript was considered as a P-wave potential. The first vertical derivative of the pressure brought us to data equivalent to the displacement and the second derivative yielded the strain proxy. Given that the simulations were performed using a 6.25 m grid we applied a box filter with four grid points to obtain an analog of 25 m GL for synthetic data which was close to one of our target real datasets. While elastic solvers routinely used in DAS data simulation and inversion, e.g., [35,36,37,38] lead to higher fidelity in DAS data amplitudes, the acoustic solver allows for computationally efficient generation of large datasets necessary for training the network.

**Figure 3:**
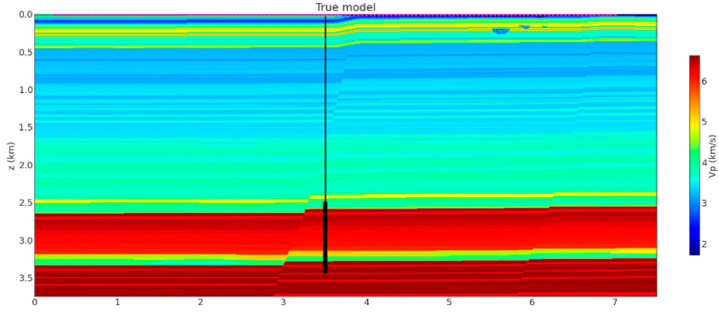
SEAM Arid slice used for data generation same as [41]. Red triangles at the top of the model mark positions of the sources used for dataset generation. DAS spans the whole well length (thin black line) while the geophones set up were focused around the target area (solid black area) leading to superior resolution.

The velocity model is based on the SEAM Arid model [39]. The SEAM Arid model is built from the Barrett model designed to represent unconventional reservoirs in Texas and near-surface model generated to represent land data challenges typically faced in exploration in the Middle East; it has been used extensively for studies on deep learning-based inversion of seismic data [40], acquisition design for VSP [41] and surface seismic [42], and evaluation of structural uncertainty in challenging desert environments [43]. We created 135 shot gathers in the size of 2000 (samples) by 1098 (traces) with different offsets ranging from 0 to 3.5 km by moving the shot location from left to right in the model in Figure 3 with increments of 50 m.

The specific parameters of forward modeling are shown in Table 2. The Klauder wavelet represents a realistic seismic vibrator sweep autocorrelation function [39]. Two widely used in seismic exploration sampling intervals of 1 and 2 ms were used for simulations. The grid size 6.25 is native to the SEAM Arid model. The central frequencies of the source between 15 and 55 Hz were picked as representing common seismic monitoring with VSP frequency content. We use the synthetic shot gathers to generate a clean set with a 512 by 512 moving window. The moving window randomly captured 20 patches from each shot gather, and finally we obtained 2700 noise-free samples. After checking that the data patches captured the first arrivals of the simulated records samples, they were randomly separated into training (70% of samples), validation (20% of samples), and testing (10% of samples) datasets.

**Figure 4:**
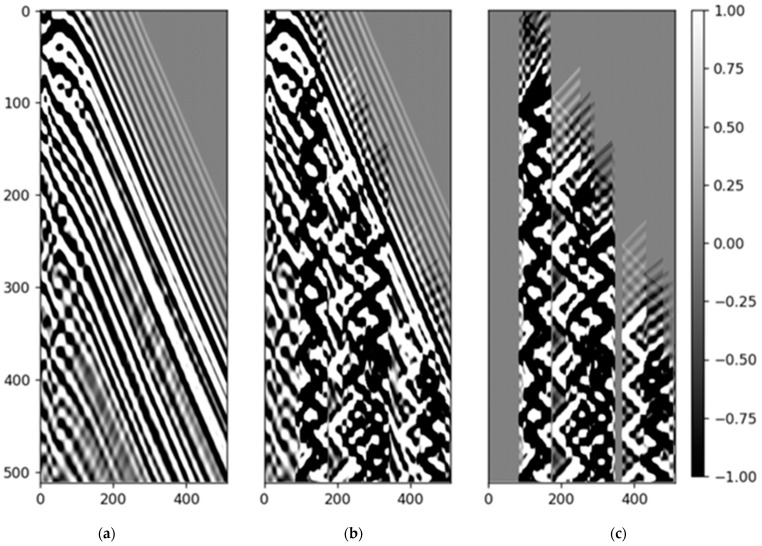
(**a**) clean data (**b**) data with noise (**c**) noise.

The generation of noise types found in field DAS-VSP records is crucial for training the diffusion model effectively. One common type of noise is the zigzag noise (N), defined by [44] as:

(10) $$N=\sum_{i=1}^{n_{max}}A_{0}x^{T_{0}+(i-1)T}*W,$$

where $T_{0}$ represents the first break time, $A_{0}$ represents the first break amplitude, T is the noise period, $n_{max}$ represents the maximum period numbers of the noise, W is the wavelet, and x represents the attenuation parameter. For synthetic data, we only considered the typical noise types which are hard to eliminate by conventional methods in DAS-VSP data, such as the zigzag noise, as shown in Figure 4c. The noise generation parameters are also specified in Table 2.

Once the clean acoustic DAS dataset was simulated, they were combined with the noise to create the synthetic dataset used for training. By injecting the zigzag noise (and possibly other types of noise) into the simulated wavefield, we created a noisy dataset that the model then learned to denoise. This process effectively trained the model to handle the kind of data that it would encounter in real-world scenarios.

## 3. Results

### 3.1. Test on the Synthetic Dataset

**Figure 5:**
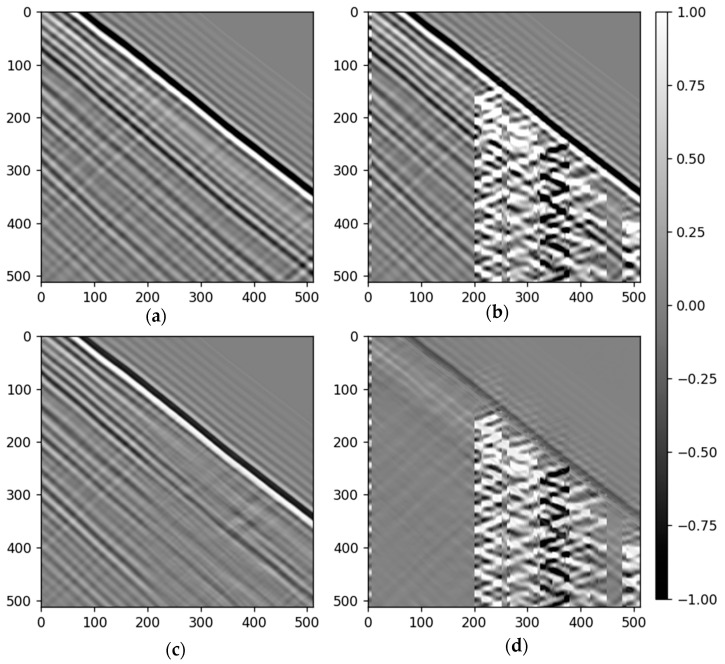
Denoising synthetic noisy data: (**a**) clean synthetic data generated using deepwave and DAS conversion. (**b**) the synthetic DAS-VSP data with noise. (**c**) the denoised result using our proposed diffusion model. (**d**) the removed noise.

To assess the efficacy of our proposed method, we initially utilized the synthetic testing dataset (Figure 5). Figure 5a displays the clean synthetic data produced via Deepwave [45] and DAS conversion. The 2D synthetic DAS-VSP data, embedded with diverse noise types, is depicted in Figure 5b, exhibiting an input SNR of 8 dB. Figure 5c illustrates the denoised outcomes derived from our proposed diffusion model, while Figure 5d portrays the extracted noise. A notable improvement in the signal-to-noise ratio (SNR) was observed, increasing to 24 dB. The denoised data gathered in Figure 5c which is barely distinguishable from the clean data in Figure 5a, and underscores our method’s capability to attenuate noise while retaining the signal. However, minor signal leakage was observed when examining the removed noise in Figure 5d. Minor signal leakage appeared to be inevitable as the signal from some upgoing reflections resembled the noise when superimposed with downgoing waves.

To further evaluate the predicted results quantitatively on the synthetic dataset, we introduced the Structural Similarity Index (SSIM) [46] as metrics. The SSIM can measure the similarity between the denoised output/noisy data and the ground truth. The SSIM ranges from −1 to 1. When two images are identical, the value of SSIM is equal to 1. The SSIM of the data shown in Figure 5, where it improved from 0.67 to 0.81.

### 3.2. Test on the Field Data

The two field DAS-VSP datasets in the study come from the Citronelle dataset [11] in the U.S., and the Groß Schönebeck site [47] in Germany. Both datasets are publicly available with more processing results available for the Groß Schönebeck site. For this study, we applied the diffusion model directly to two field DAS-VSP datasets for denoising. While the diffusion model itself was trained using synthetic data, this represents the first demonstration of its application to real-world DAS-VSP data for noise removal.

The Citronelle DAS-VSP dataset was acquired in Citronelle Field, Alabama in 2016. The test site is a Triassic fluvial-dominated sandstone reservoir located at approximately 10,000 feet depth. The dataset was obtained using DAS technology along two ~1 km deep vertical monitor wells with a VSP geometry. Controlled injection of ~22,000 tons of CO_2_ created a CO_2_ plume extending ~300 m. The high-resolution DAS-VSP dataset allows detailed imaging and characterization of the reservoir architecture, fracture networks, fluid distributions, and CO_2_ plume behavior before, during, and after injection. This dataset provides an invaluable resource to study time-lapse subsurface changes associated with CO2 injection in a geologic reservoir using DAS-VSP.

**Figure 6:**
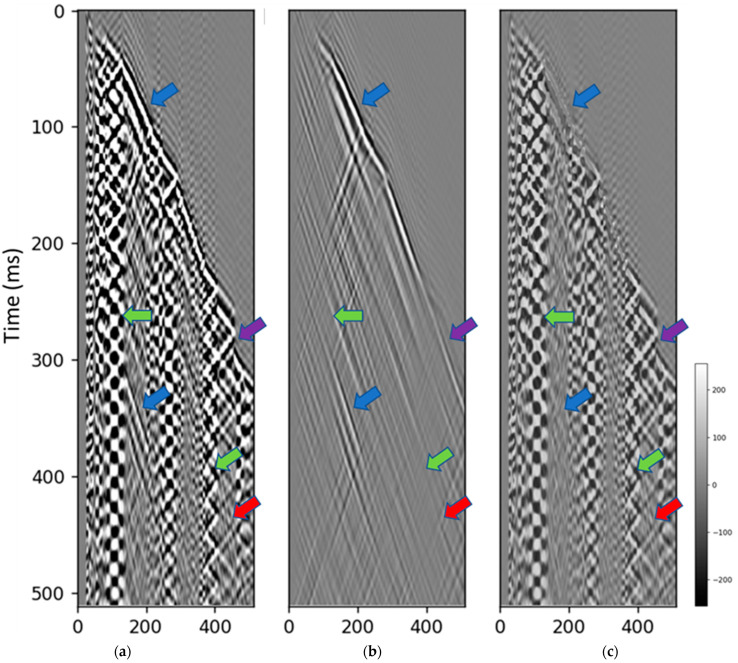
The denoising result for the Citronelle dataset. (**a**) field data input, (**b**) diffusion model denoised data, (**c**) predicted noise. The green arrows indicate that the noise is successfully suppressed. The blue arrows indicate the preserved signals. The purple arrow indicates the calibration of first arrival. The red arrow indicates the signal leakage.

Comparisons between the input data shown in Figure 6a, denoised output (Figure 6b), and predicted noise (Figure 6c) provide several key observations. The green arrows indicate the areas where the noise was successfully suppressed in the denoised output, compared to the raw input data. This included attenuation of strong zigzag and checkerboard noise patterns. The blue arrows pinpoint the regions where primary reflection signals were preserved during denoising. The continuity and relative amplitudes of these events were maintained from input to output. The purple arrow shows an example where the first arrival direct wave was properly calibrated and aligned between the input and denoised data. This demonstrates the model’s ability to retain important signal components. The red arrow highlights an area of signal leakage where remnants of the reflection event persisted in the predicted noise. This indicates the model needs further tuning to completely remove noise without signal loss.

The Groß Schönebeck area is part of a large geothermal project in Germany where 4.2 km deep wireline DAS was acquired for the first time [47] which after major efforts in processing [48] led to successful imaging results in 3D VSP setup [49]. Public data retrieved for Groß Schönebeck dataset include correlated stacked noisy data and data after adaptive deconvolution.

**Figure 7:**
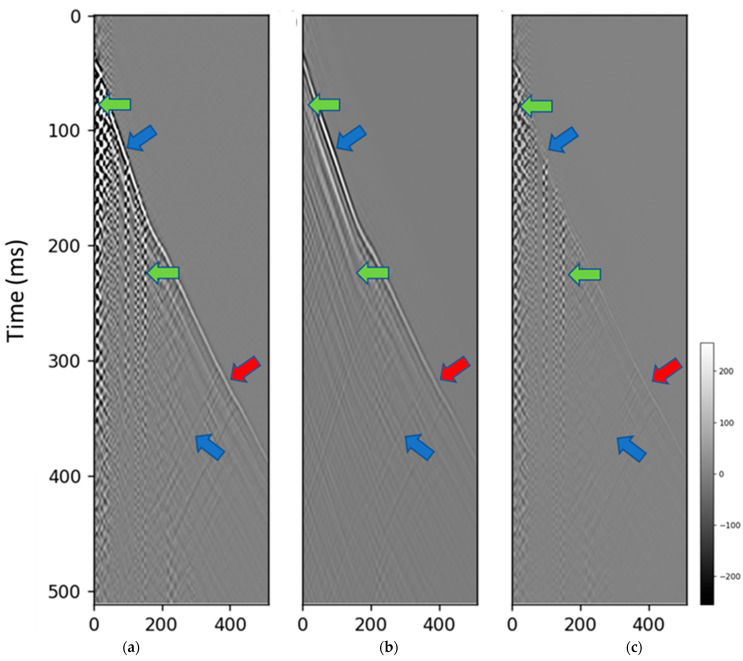
The denoising result for the Groß Schönebeck dataset. (**a**) field data input, (**b**) diffusion model denoised result, (**c**) predicted noise. The green arrows indicate that the noise is successfully suppressed. The blue arrows indicate the preserved signals. The red arrow indicates the signal leakage.

**Figure 8:**
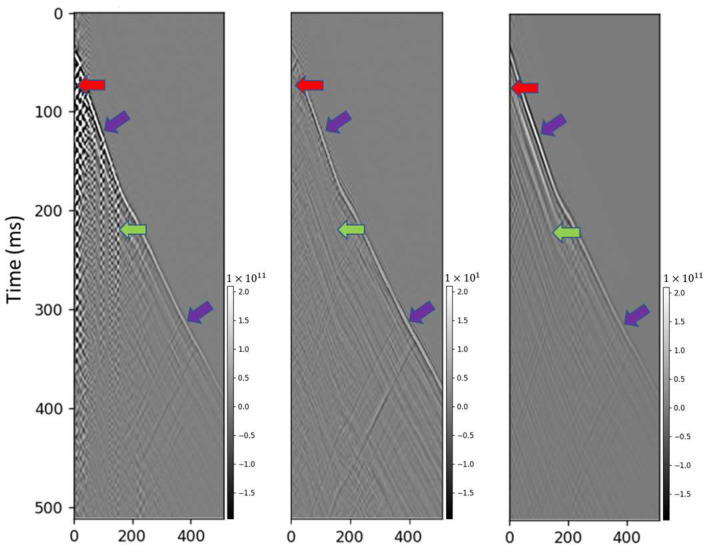
Comparison with deconvolution. (**a**) field data input, (**b**) adaptive deconvolution result, (**c**) diffusion model denoised result. The red and green arrows indicate noisy areas in the data. The green arrow indicates the area where denoising is successful in both methods. The red arrow indicates that the deconvolution does not mitigate the noise, but the diffusion model does. The purple arrows indicate that the deconvolution changes the amplitude which the diffusion model preserves.

**Figure 9:**
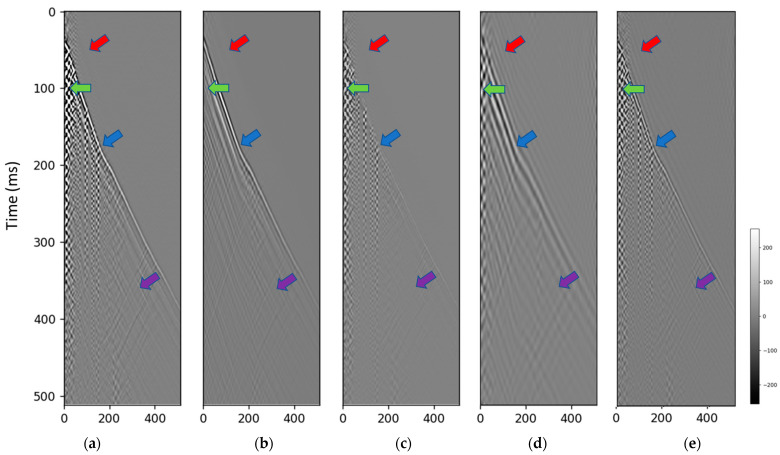
Comparison with bandpass filter. (**a**) field data input, (**b**) diffusion model denoised result, (**c**) residual between (**a**,**b**), (**d**) bandpass filter result, (**e**) residual between (**a**,**d**). The red arrow indicates aliasing introduced by the bandpass filter. The blue arrow points out the elimination of the first arrival by the bandpass filter, while it is preserved by the diffusion model. The green arrow denotes that the bandpass filter does not mitigate the noise, in contrast to the diffusion model. The purple arrow highlights that the bandpass filter induces significant signal leakage and reduces the resolution.

The diffusion model removed the noise even in this case allowing for picking around the top of the well which significantly simplified the subsequent processing for the near-surface data (Figure 7). Adaptive deconvolution is aimed at making data sparser and spectrum flat is performed trace-by-trace and results into substantial amplitude changes and disbalancing, yet removes a significant portion of the zigzag noise caused by cable reverberations [48]. For the deeper part of the well, the reverberations were significantly slower than the direct arrivals and spatially separated and deconvolution worked well. However, deconvolution failed in the case when the reverberations had comparable speed to the first arrivals and were spatially not separated from the direct arrivals (Figure 8); this was particularly detrimental to the top part of the well that could not be picked due to intersecting events (red arrow in Figure 8b). To further assess the results generated by the proposed method, we applied a bandpass filter to extract a higher SNR bandwidth (10–30 Hz) to the Groß Schönebeck dataset and juxtaposed the filtered data with those derived from the diffusion model (Figure 9). While the bandpass filter result (Figure 9d) did partially remove some of the zigzag noise, it also removed a significant portion of signal introducing several issues including reduced temporal resolution (Figure 9e).

## 4. Discussion

### 4.1. Diffusion Time-Step Analysis

Diffusion models function by simulating a forward diffusion process that begins from a target distribution—in this study, the noise-free DAS-VSP samples—and gradually infuses noise until reaching a simple distribution, such as a standard Gaussian distribution. This forward process is computationally straightforward, but the reverse procedure of transitioning from the simple to the target distribution is more intricate and requires iterative training.

The diffusion process is typically partitioned into discrete timesteps. The quantity of the timesteps can significantly influence the model performance. We investigated the impact of various timestep numbers on the efficacy of our proposed diffusion model, ranging from 1 to 1000.

A single timestep (T = 1) configuration essentially requires the diffusion model to encapsulate the entire distribution in one instance. This task is substantial as the transformation from the simple to the target distribution is highly complex, leading to the potential for distortion or anomalies in the output. When the timestep quantity is set to 1 for both forward and reverse diffusion processes, the model assumes the role of a single step denoising model, adding and then trying to recover noise in one action. The U-Net, in this case, serves as the architecture for the reverse process, tasked with mapping the noisy data back to the original data. While the diffusion process is simplified, the U-Net’s task is more intricate, given that it must reverse the noise effects in one go. Hence, the diffusion model with a single timestep could be viewed as a single-step denoising U-Net.

Introducing 10 timesteps (T = 10) allows the diffusion model more ‘room’ to progressively transform the simple distribution into the target distribution. While the sample quality may improve compared to a single timestep, the output could still vary noticeably from the target distribution. This situation signifies a trade-off between computational complexity and sample quality.

Expanding to 100 timesteps (T = 100), the model can facilitate more gradual transitions from the simple to the target distribution. Consequently, the quality of the generated samples could surpass those of models with fewer timesteps. However, the increased computational requirement and potential risk of overfitting are important considerations.

**Figure 10:**
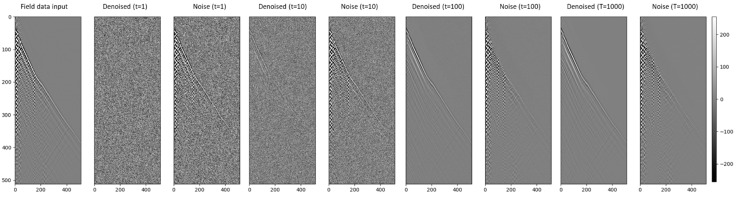
Diffusion process in different timestep.

Finally, with 1000 timesteps (T = 1000), the model is provided ample opportunity to make very incremental transitions from the simple to the target distribution. The generated samples could potentially be of high quality, virtually indistinguishable from the target distribution. But the computational expense escalates significantly, and careful regularization could be necessary to circumvent overfitting. Figure 10 shows the diffusion process with T = 1000. The noise is gradually removed with timestep increasing.

There exists a trade-off between the settings of timesteps and computational efficiency. For simpler tasks, a smaller timestep may suffice. Certain derivatives of diffusion models, like the deep diffusion implicit model (DDIM) by [50], can achieve comparable performance with smaller timesteps as they would with larger ones.

### 4.2. Advantages and Limitations

The injection of noise at every timestep can be considered a form of data augmentation. This process adds varying levels of noise to each training image at each timestep, creating an altered version for the model to learn from. This inherent data augmentation property can prove beneficial, especially when working with a limited dataset.

Comparisons with classic methods for denoising for DAS data described here are limited to the deconvolution and bandpass filter methods, but generally machine learning based methods outperform classic denoising methods as multiple recent studies suggest [15,19].

While diffusion models have several advantages, such as robustness to varying noise levels and an ability to handle complex distributions, they also pose challenges like increased computational demands. This increased complexity stems from the sequential nature of the diffusion process, compared to models like the U-Net. Furthermore, the quality of results still heavily depends on the training data quality and variety in supervised learning applied to train the diffusion models.

The computational expense of the current diffusion model implementation poses a potential limitation, particularly due to the large timestep setting. At the current stage, inference for a single shot gather takes about a minute while training requires several days. However, there are ways to reduce the training and inference time requirements by using transfer learning and adopting more computationally efficient diffusion model architectures actively researched in the machine learning community.

## 5. Conclusions

We proposed a DAS-VSP denoising workflow based on the conditional diffusion model, which was trained on a relatively small synthetic DAS-VSP shots dataset. The model’s performance was evaluated using synthetic and field DAS-VSP datasets. When compared to traditional methods, the proposed model offers a more robust and general solution for suppressing typical noise in DAS-VSP data.

The field of generative modeling is currently undergoing rapid and significant advancements. It is our hope that this pioneering research contributes to the ongoing development of this field, paving the way for further applications of the generative modeling techniques, such as diffusion models, in seismic and DAS-related research.

## Figures and Tables

**Table 1:** Detailed hyperparameters in the diffusion model.

Hyperparameters	Value
Patch size	512 × 512
Kernel size	3 × 3
Batch size	64
Epoch	500,000
Initial learning rate	${2\times 10}^{-5}$

**Table 2:** Specific parameters of forward modeling and noise generation.

Parameters	Value
Wavelet	Klauder
Time interval (s)	0.001, 0.002
Grid size (m)	6.25
Boundary condition	PML
Frequency [min, max] (Hz)	[15, 55]
Maximum period [min, max] ($n_{max}$)	[20, 40]
Noise attenuation parameter [min, max] (x)	[0.05, 0.7]

## Data Availability

The Citronelle DAS VSP dataset can be found in https://edx.netl.doe.gov/dataset/citronelle-2013-das-vsp. The Groß Schönebeck dataset can be found in https://doi.org/10.5880/GFZ.4.8.2021.001. The Deepwave can be found in https://doi.org/10.5281/zenodo.8189232.
